# The Oncolytic Effect of Aerva lanata on Osteosarcoma Cell Lines via the Apoptotic Signaling Pathway

**DOI:** 10.7759/cureus.58091

**Published:** 2024-04-12

**Authors:** Ummu Zuvairiya, Menaka S, Selvaraj Jayaraman, Vasugi Suresh

**Affiliations:** 1 Department of Physiology, Saveetha Dental College and Hospitals, Saveetha Institute of Medical and Technical Sciences, Saveetha University, Chennai, IND; 2 Centre of Molecular Medicine and Diagnostics (COMManD), Department of Biochemistry, Saveetha Dental College and Hospitals, Saveetha Institute of Medical and Technical Sciences, Saveetha University, Chennai, IND

**Keywords:** p53 gene, bcl-2 mrna, gene expression, apoptosis, oncolytic effect, saos2 cell lines, osteosarcoma, aerva lanata

## Abstract

Introduction

Osteosarcoma, a malignant bone tumor, poses significant treatment challenges, necessitating the development of alternative therapeutic strategies. *Aerva lanata* (*A. lanata),* a medicinal plant with traditional use in various healthcare systems, has anti-cancer properties. This study looks at the oncolytic effect of *A. lanata* extract on osteosarcoma cell lines (sarcoma osteogenic-Saos2).

Aim

The aim of this study was to investigate the oncolytic effect of *Aerva lanata* on Saos2 cell lines through the apoptotic signaling pathway.

Materials and methods

*A. lanata* extract was prepared using Soxhlet extraction, and its cytotoxic effects on Saos2 cells were assessed using the 3-(4,5-dimethylthiazol-2-yl)-2,5-diphenyltetrazolium bromide (MTT) assay. Real-time polymerase chain reaction (RT-PCR) analysis of gene activity was used to assess the extract's effect on apoptotic signaling pathways.

Results

The MTT assay demonstrated a dose-dependent decrease in Saos2 proliferation following treatment with *A. lanata* extract at concentrations ranging from 50 μg to 200 μg. The standard deviations observed ranged from 1.414 to 7.071. Gene expression analysis revealed that the extract led to a reduction in the messenger ribonucleic acid (mRNA) levels of the anti-apoptotic marker B-cell lymphoma 2 (Bcl2), with standard deviations ranging from 1 to 0.535. Conversely, it induced an increase in the mRNA levels of the tumor suppressor protein p53, with standard deviations ranging from 1 to 1.835. These findings suggest that the extract modulates the apoptotic pathways of the Bcl2 and p53 genes.

Conclusion

*A. lanata* extract exhibits promising anti-cancer activity against Saos2 osteosarcoma cell lines, inducing apoptosis by downregulating Bcl2 and increasing p53. The study's findings suggest that *A. lanata* may be useful as a natural treatment for osteosarcoma.

## Introduction

Osteosarcoma, a primary malignant bone tumor predominantly affecting adolescents and young adults, poses significant challenges in oncology due to its aggressive nature and limited treatment options [1]. Despite advancements in therapeutic modalities, including surgery, chemotherapy, and radiation, the prognosis for osteosarcoma patients remains relatively poor, especially in cases of metastatic or recurrent disease. Thus, it is imperative to investigate cutting-edge therapy strategies that might effectively target osteosarcoma cells while minimizing detrimental effects on healthy tissue. The curative efficacy of natural medicines made from medicinal plants in the treatment of cancer appears to have piqued interest in recent decades. *Aerva lanata* (*A. lanata*), commonly known as Mountain Knotgrass, is one such plant that has been extensively researched for its medicinal benefits, particularly its anti-cancer effects. *A. lanata* has a long tradition of use in conventional medical systems, such as Ayurveda and Siddha, where it is utilized for its diverse medicinal properties. The rationale for investigating *A. lanata* as a potential therapeutic agent against osteosarcoma lies in its rich phytochemical composition, which includes alkaloids, flavonoids, tannins, saponins, and terpenoids, among others [2]. These bioactive substances have demonstrated a variety of biological activities, including anti-cancer, antioxidant, anti-inflammatory, and anti-bacterial qualities. Importantly, several preclinical studies have provided compelling evidence supporting the anti-cancer activity of *A. lanata* against different kinds of cancer, including prostate, lung, breast, and colon cancer [3]. Extracts from *A. lanata* exhibit cytotoxic effects on cancer cell lines like Michigan Cancer Foundation-7 (MCF-7) and human liver cancer cell line (HepG2), potentially due to the bioactive compounds present. Moreover, its ability to cause cell death in several cancer cell lines, including PC-3, HeLa, and HT-29, emphasizes its function in eradicating aberrant cells [4]. Furthermore, the anti-inflammatory properties of *A. lanata* may aid in suppressing inflammation-associated tumorigenesis, thus contributing to its potential as an anti-cancer agent. However, its efficacy against osteosarcoma, particularly using osteosarcoma cell lines (sarcoma osteogenic-Saos2), remains relatively unexplored [5]. 

Saos2 cell lines are widely used as in vitro models for studying osteosarcoma due to their high proliferative capacity and genetic stability. Saos2 cell lines are derived from a human osteosarcoma biopsy obtained from a 10-year-old Caucasian female. They were initially established in 1973 by Fogh and Trempe at the Memorial Sloan-Kettering Cancer Center in New York. Saos2 cells are defined by their ability to form multicellular aggregates and to produce tumors when implanted into immunocompromised mice, reflecting their malignant nature [6]. Saos2 cells additionally produce osteoblastic differentiation markers like alkaline phosphatase and osteocalcin. This makes them useful for researching the molecular mechanisms underlying osteoblastic differentiation and bone formation, as well as studying diseases like osteosarcoma [7]. In addition to their genetic stability and high proliferative capacity, Saos2 cells are amenable to various genetic manipulations, such as transfection and gene knockout, making them versatile for studying gene function and signaling pathways involved in osteosarcoma development and progression [8]. Furthermore, Saos2 cells have been extensively utilized in preclinical drug screening assays to evaluate potential therapeutic agents for osteosarcoma treatment. Their ability to respond to chemotherapeutic drugs like doxorubicin and cisplatin, which are frequently used to treat osteosarcoma, emphasizes the value of using them as an in vitro model for drug development [9].

Evaluating the anti-cancer activity​​​​​​​ of *A. lanata* against Saos2 cell lines was the aim of the investigation. Considering the urgent requirement for safe and efficient osteosarcoma therapy options, it has great potential. *A. lanata* extract may provide a novel therapeutic approach that enhances current treatment modalities by focusing on important molecular pathways implicated in the onset and progression of osteosarcoma.

## Materials and methods

Chemicals 

Gibco, Canada provided the following reagents: trypsin-EDTA, fetal bovine serum (FBS), antibiotics-antimycotics, Dulbecco's modified Eagle's medium (DMEM), and phosphate-buffered saline. Invitrogen, USA also provided JC-1 (5,5,6,6-tetrachloro-1,1,3,3-tetraethyl benzimidazole carbocyanine iodide) and a real-time polymerase chain reaction (RT-PCR) kit (MESA Green). All chemicals used were of analytical grade and extra pure.

Extract preparation

One hundred grams of *Aerva lanata* leaf powder was extracted using 500ml of 70% ethanol solution through a Soxhlet apparatus. The mixture went through Whatman No. 1 filtering sheets following separation. A viscous mass was then produced by evaporating the solvent using a rotary evaporator apparatus at a lower pressure. After that, this bulk was kept in storage at 4 °C until it was needed for further use.

Procurement and culture of osteosarcoma cells

Saos2 cells, obtained from the American Type Culture Collection, Manassas, VA, were cultured in DMEM supplemented with 10% FBS. The cell cultures were maintained at 37 °C in a 5% CO_2_-humidified atmosphere. Saos2 cells were isolated and initially cultured in DMEM supplemented with 10% FBS and 100 U/mL penicillin for one week.

Cytotoxicity studies

Following the plating of 5 x 10^5^ Saos2 per well in 96-well plates, the cells were allowed to attach overnight. After attachment, the cells were treated in triplicate with varying concentrations of Bixa orellana bark extracts and incubated for 24 hours at 37 °C in a humidified atmosphere with 5% CO_2_. Subsequently, the 3-(4,5-dimethylthiazol-2-yl)-2,5-diphenyltetrazolium bromide (MTT) reagent was added to each well. The formazan that was generated was dissolved in dimethyl sulfoxide (DMSO) following an additional four hours of incubation. The optical density (OD) of the solutions was determined using a spectrometer, which has a wavelength of 570 nm. Three independent trials were conducted to ascertain the mean OD ± standard deviation (SD) for each group. Using the following formula, the inhibitory rate of cell growth was determined:

% Growth inhibition = (1 - OD_extract treated) / (OD_negative control) x 100.

Methods for cell sustainability

In 96-well plates, 5000 cells per well of Saos2 were seeded. The plates were then incubated for 48 hours before being subjected to tussilagone (TSL) and gallic acid fractions. After that, the MTT test was carried out by adding MTT solution to the cells and using a microplate reader to measure absorbance at 540 nm [10].

Propidium iodide double-stained flow cytometry with Annexin V coupled with fluorescein isothiocyanate (V-FITC)

Saos2 cells were planted at a density of 2×10^5^ cells per well in six-well plates. Following different lengths of TSL-1 exposure, cell populations were isolated, stained with the Annexin V-FITC Apoptosis Detection Kit, and examined using a laser flow cytometer.

Gene expression evaluated by RT-PCR

To ascertain the amount of mRNA expression, RT-PCR was employed. Total RNA was extracted with Tri Reagent, then reverse transcribed using a Superscript III first-strand complementary deoxyribonucleic acid (cDNA) synthesis kit. The PCR reactions were conducted using the MESA Green PCR master mix. The comparative cycle threshold (CT) approach was utilized to evaluate the data, and the 2^(-ΔΔCT) method was employed to calculate the fold change. The 2^(-ΔΔCT) is a methodology that compares threshold cycle (CT) values adjusted to housekeeping genes in order to evaluate changes in gene expression compared to controls. The amplification product's specificity was verified by a melting curve examination. Glyceraldehyde 3-phosphate dehydrogenase (GAPDH) served as the standard gene [11].

Analytical statistics

Three separate tests, each carried out in triplicate, produced the data, which were shown as means ± SD. The one-way analysis of variance (ANOVA) was used for the statistical analysis, and p < 0.05 was the significance level.

## Results

**Figure 1:**
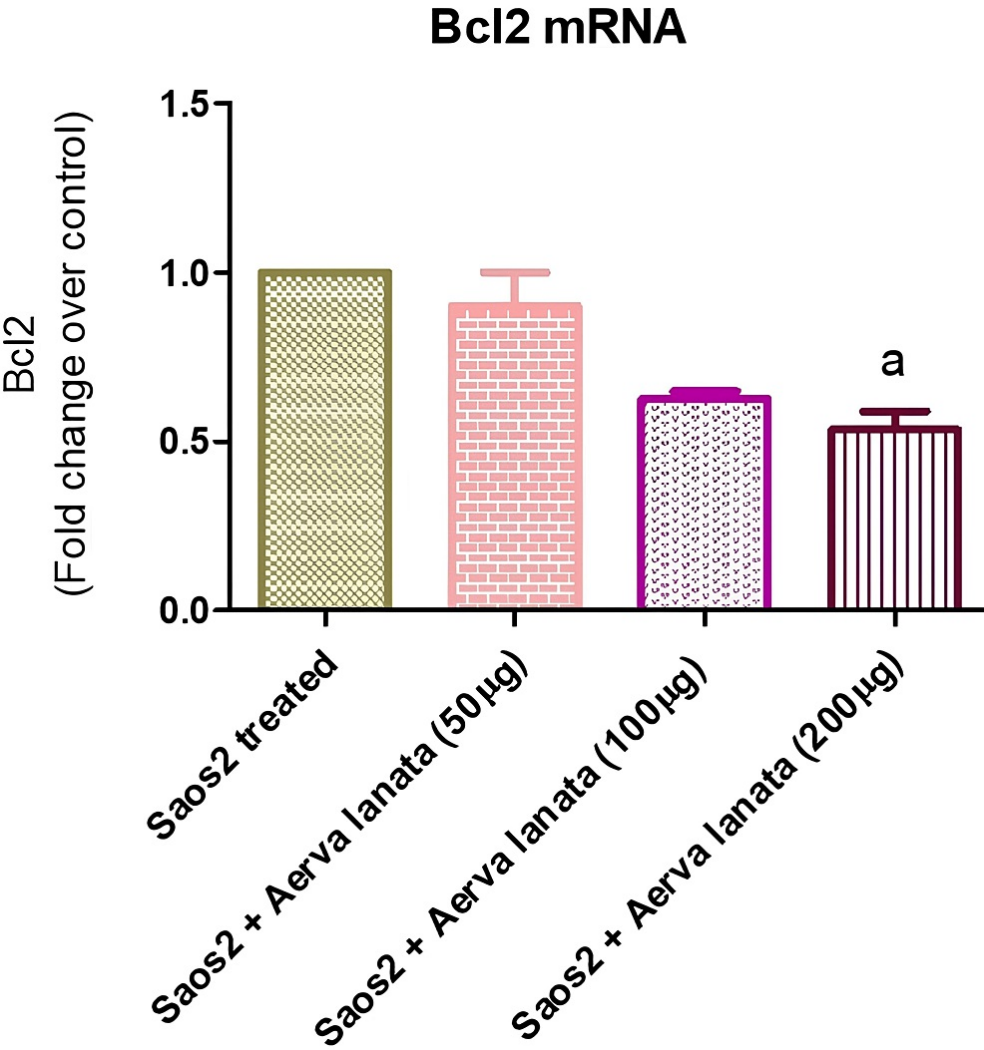
Bcl2 mRNA Gene Downregulation in Saos2 Cell Lines Treated With Various Concentrations of A. lanata Bcl2: B-cell lymphoma 2; mRNA: messenger ribonucleic acid; Saos2: osteosarcoma cell lines; *A. lanata*: *Aerva lanata*

Figure 1 presents the B-cell lymphoma 2 (Bcl2) messenger ribonucleic acid (mRNA) gene expression levels in both the control and treated groups with varying concentrations (50 μg, 100 μg, and 200 μg) of *A. lanata*. Bcl2 mRNA encodes the Bcl2 protein, known for its role in inhibiting apoptosis and promoting cell survival. Dysregulation of Bcl2 expression or function is implicated in various diseases, including cancer, where overexpression of Bcl2 can confer increased cell survival and resistance to apoptotic stimuli. The mean expression levels observed in the treated groups at concentrations 1, 0.625, and 0.535 are 0.9005, 0.625, and 0.535, respectively, compared to the control. The standard deviation and standard error values provide insight into the variability and precision of these measurements. These results suggest a potential modulatory effect of *A. lanata* on Bcl2 mRNA expression, which may have implications for cell survival and apoptotic pathways, particularly in the context of diseases like cancer.

**Figure 2:**
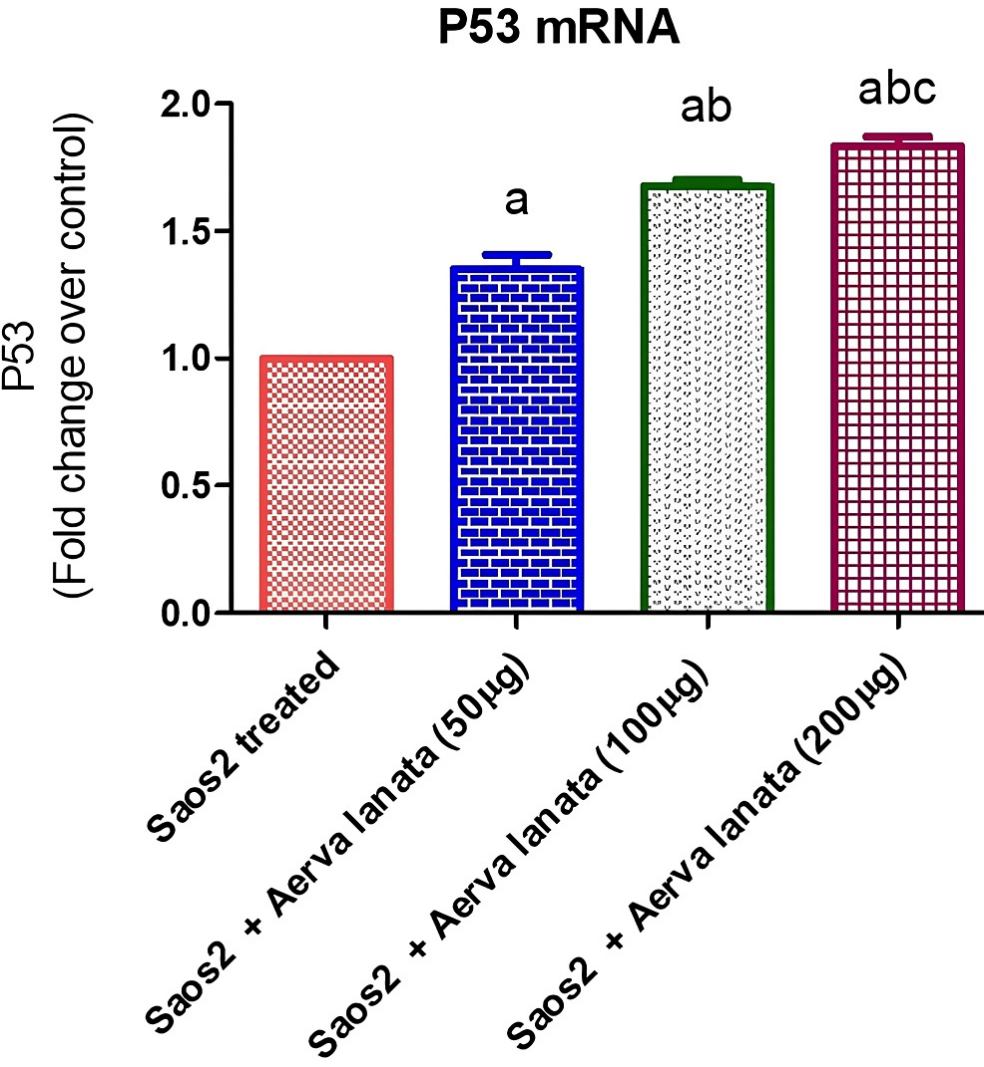
Activation of p53 Gene Expression in Saos2 Cell Lines Treated with Various Concentrations of A. lanata p53: Tumor suppressor protein; Saos2: osteosarcoma cell lines; *A. lanata*: *Aerva lanata*

Figure 2 illustrates the expression levels of the tumor suppressor protein (p53) gene in response to escalating concentrations of *A. lanata* extract. The p53 gene acts as a tumor suppressor, playing a crucial role in halting tumor formation. As depicted, there is a noticeable increase in the expression of the p53 gene with higher concentrations of the *A. lanata* extract. The mean expression levels observed at concentrations 1, 1.677, and 1.835 are 1.349, 1.677, and 1.835, respectively, compared to the control group. Standard deviation and standard error values provide insights into the variability and precision of these expression measurements. These findings suggest that *A. lanata* extract may have a stimulatory effect on p53 gene expression, which could potentially contribute to its tumor-suppressive properties.

**Figure 3:**
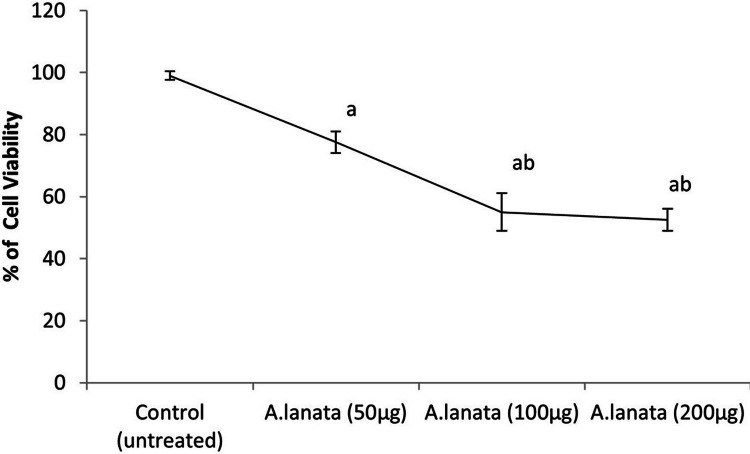
Cell Viability Percentage in Control and A. lanata Treated Saos2 Cell Line as Determined by MTT Assay *A. lanata*: *Aerva lanata;* Saos2: osteosarcoma cell lines; MTT: 3-(4,5-dimethylthiazol-2-yl)-2,5-diphenyltetrazolium bromide

Figure 3 depicts the impact of varying concentrations of *A. lanata* extract on the cell viability of Saos2 cell lines, measured as the percentage of viable cancerous cells using MTT assay. As the concentration of the extract increases incrementally, a noticeable trend emerges: the cell viability of cancerous cells decreases correspondingly. Specifically, at the concentrations provided, the mean cell viability is 89%, 69%, 54.5%, and 45%, respectively. The standard deviation, ranging from 1.414 to 7.071, indicates the degree of variability within each concentration group, while the standard error, ranging from 1 to 5, reflects the precision of the mean estimates. These values suggest a consistent decline in cell viability with increasing extract concentrations. The results suggest a dose-dependent effect of *A. lanata* extract on reducing the viability of cancerous cells.

## Discussion

The anti-cancer activity of *A. lanata* against Saos2 has been investigated through multiple assays, including the MTT assay, Bcl2 mRNA expression analysis, and p53 gene mRNA expression analysis. The findings from these assays provide valuable insights into the potential therapeutic effects of *A. lanata* in combating osteosarcoma. To begin, increasing *A. lanata* extract concentrations leads to a dose-related decrease in cell survival, as measured by the MTT assay. This suggests that the extract has cytotoxic effects on Saos2 cells, implying that it could be used as a treatment for osteosarcoma [12].

Previous research found that silver-copper (Ag-Cu) nanoparticles extracted from *A. lanata* have a significant cytotoxic impact on malignant HeLa cell lines, with an IC50 of 17.63 µg/mL. They possess powerful antioxidant effects and may decrease and scavenge H_2_O_2_ radicals at elevated levels (240 µg/mL). These findings highlight the significant potential of *A. lanata* plant metabolite-driven nanoparticles as effective cancer therapies [13]. The MTT assay, which assesses the viability of osteosarcoma cell lines against *A. lanata*, provides valuable information about its therapeutic potential [14]. Furthermore, when compared to conventional chemotherapy agents such as doxorubicin and cisplatin, *A. lanata* exhibits comparable or superior cytotoxic effects against osteosarcoma cells, with lower toxicity to normal cells. Understanding the mechanisms that underpin its anticancer activity in comparison to other natural products or conventional drugs can shed light on its possibility as an innovative therapeutic option [15].

Moreover, Bcl2 mRNA expression investigation showed a noteworthy reduction in Bcl2 mRNA levels in response to *A. lanata *treatment [16]. It is well recognized that Bcl2 is essential for enhancing cell survival and preventing apoptosis. The downregulation of Bcl2 mRNA gene expression by *A. lanata* extract may be attributed to its phytochemical composition, such as cardiac glycosides like digitoxin and gitoxin, which have been shown to induce apoptosis by inhibiting Bcl2 expression. Additionally, flavonoids present in *A. lanata*, such as quercetin and kaempferol, can downregulate Bcl2 expression through various signaling pathways involved in apoptosis. Moreover, sesquiterpene lactones like helenalin found in *A. lanata* may disrupt Bcl2 function, promoting apoptotic cell death in cancer cells [17]. Several studies have reported similar findings, demonstrating that natural extracts or compounds can modulate Bcl2 expression, resulting in apoptotic cell death in cancer cells [18]. Other herbal extracts or phytochemicals, such as curcumin, resveratrol, and green tea polyphenols, have been shown in studies to reduce Bcl2 expression and cause apoptosis in multiple types of cancer cells, such as osteosarcoma [19]. This consistent pattern emphasizes the significance of targeting Bcl2 as a therapeutic strategy against cancer, including osteosarcoma, and suggests that *A. lanata* may have potential as a natural agent for Bcl2-targeted therapy in this setting [20]. Additional investigation into the precise processes behind *A. lanata*'s modification of Bcl2 and its effectiveness in relation to other natural chemicals may yield important information for the creation of novel anti-cancer treatments for patients with osteosarcoma. Thus, potential of *A. lanata* to cause apoptosis in Saos2 cells is suggested by the downregulation of Bcl2 mRNA expression, which lends additional credence to its anti-cancer properties [21].

Additionally, the expression analysis of the p53 gene revealed an increase in p53 mRNA levels upon treatment with *A. lanata* extract. A gene known as p53 inhibits cancer by controlling DNA repair, the stoppage of the cell cycle, and the death of cells [22]. The upregulation of p53 gene expression suggests that *A. lanata* may activate p53-mediated apoptotic pathways, contributing to its anti-cancer effects against osteosarcoma [23]. Comparisons with previous research revealed similar trends in natural compound-mediated modulation of p53 expression in cancer cells, albeit with methodological differences [24]. The observed upregulation of p53 is consistent with its tumor-suppressive function, implying that *A. lanata* is a promising anti-cancer agent that promotes tumor suppressive pathways. More research into the molecular mechanisms and downstream effects of *A. lanata* on p53 signaling pathways is needed to fully understand its therapeutic potential in osteosarcoma treatment [25]. This work adds to the increasing amount of research that supports the use of natural chemicals in cancer treatment. It highlights the importance of further research into the potential of *A. lanata* as a therapy for osteosarcoma [26]. In general, the outcomes of these assays support the hypothesis that *A. lanata* has anti-cancer activity against Saos2. The extract induces cytotoxic effects, potentially through the modulation of Bcl2 and p53 gene expression, leading to apoptotic cell death [27].

**Figure 4:**
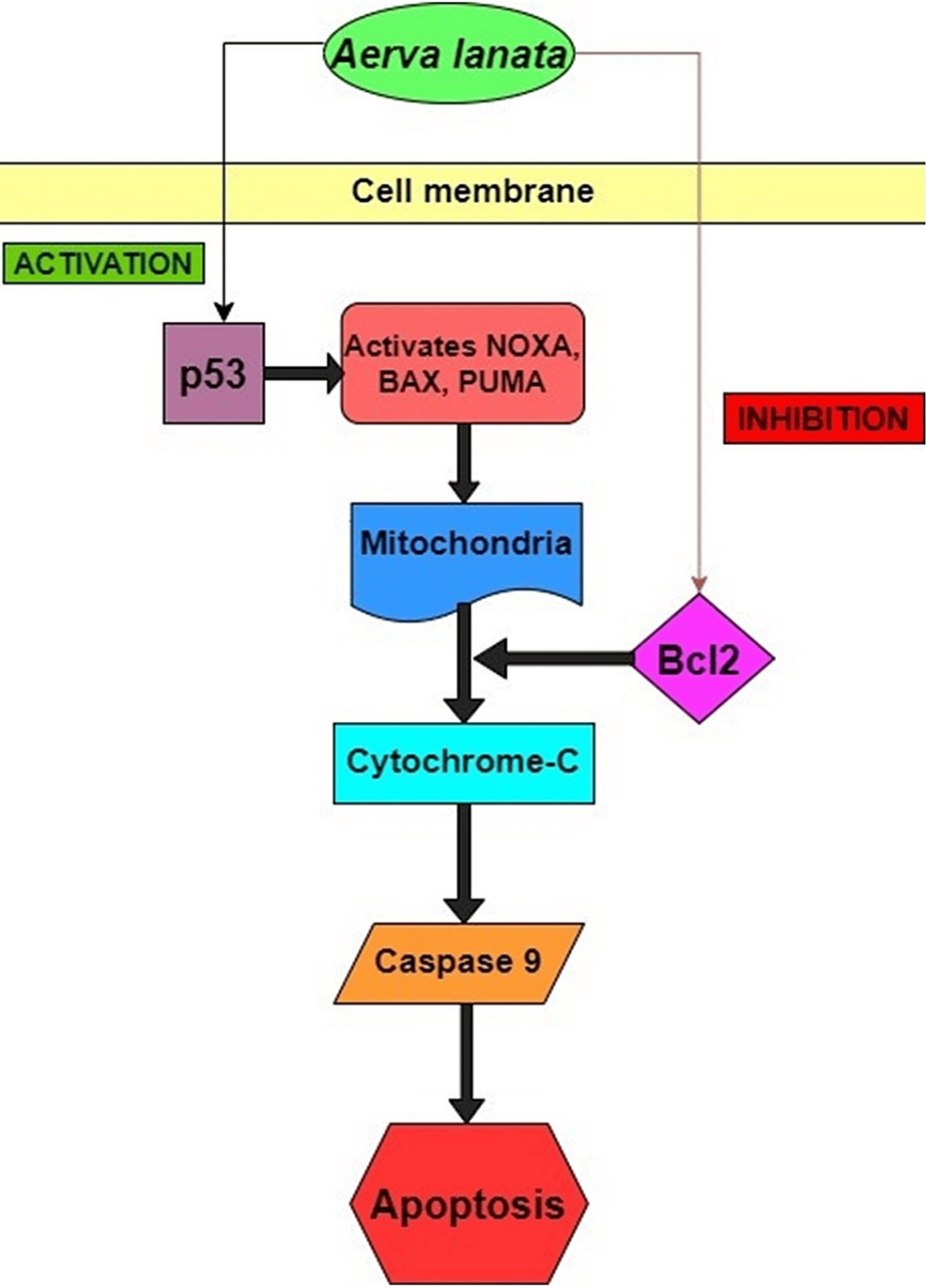
Comprehensive Apoptosis Pathway Involving p53 and Bcl2 Genes in the A. lanata-Treated Saos2 Cell Line p53: tumor suppressor protein, PUMA - p53 upregulated modulator of apoptosis, NOXA - Nicotinamide Adenine Dinucleotide Phosphate (NADPH) Oxidase Activator, Bcl2: B-cell lymphoma2, BAX - Bcl2 Associated X, *A. lanata: Aerva lanata*,  Saos2: osteosarcoma cell lines Image Credit: Menaka S

Figure 4 illustrates an overview of the Apoptosis Pathway Involving p53 and Bcl2 Genes in *A. lanata* Treated Saos2 Cell Line. These findings underscore the need for further investigation into the molecular mechanisms underlying anti-cancer properties of A. lanata, suggesting its potential as a therapeutic agent for osteosarcoma treatment.

Limitation

The study primarily focuses on in vitro experiments, which may not fully capture the complexities of in vivo tumor microenvironments. In the future, the research will extend to in vivo studies to assess the effect of *A. lanata* on osteosarcoma patients.

## Conclusions

Finally, our research results deliver overwhelming proof that *A. lanata* extract has anticancer properties against Saos2. We found that the extracts had cytotoxic effects on Saos2 cells, as evidenced by a dose-dependent decline in cell viability as the extract concentrations rose using the MTT test. Furthermore, our findings revealed significant modulation of both Bcl2 mRNA expression, which is linked to apoptotic pathways, and p53 gene mRNA expression, which is a key tumor suppressor gene. These findings suggest that *A. lanata* extract may have anti-cancer properties via mechanisms involving apoptosis regulation and tumor suppression. To confirm its therapeutic promise in treating osteosarcoma, more investigation into the underlying molecular pathways and in vivo investigations are needed.
